# Tendon of Flexor Carpi Radialis in carpal tunnel: a radiologic and cadaveric study

**DOI:** 10.3906/sag-2012-31

**Published:** 2021-08-30

**Authors:** Burcu ERÇAKMAK GÜNEŞ, Alper VATANSEVER, Deniz DEMİRYÜREK, Mine ERGUN, Hakan ÖZSOY

**Affiliations:** 1 Department of Anatomy, Faculty of Medicine, Hacettepe University, Ankara Turkey; 2 Department of Anatomy, Faculty of Medicine, Balıkesir University, Balıkesir Turkey; 3 Department of Orthopedy and Traumatology, Memorial Hospital, Ankara Turkey

**Keywords:** Cadaver, carpal tunnel, flexor carpi radialis, magnetic resonance imaging, wrist anatomy

## Abstract

**Background/aim:**

Carpal tunnel is an important anatomical passage that carries the flexor tendons into the hand. As there is still no consensus about its contents among the anatomy textbooks, the main purpose of this study was to identify the relations of the flexor carpi radialis tendon in the carpal tunnel.

**Materials and methods:**

This retrospective study was completed in April 2018 at authors’ university’s hospital. Seventy-four female and 44 male patients’ wrists without any pathology were examined by using magnetic resonance images. The series of axial sections where the pisiform exist were evaluated by using T1 sequence and the structures in the carpal tunnel were identified.

**Results:**

Results of this study showed that the tendon of the flexor carpi radialis was found above the flexor retinaculum within its own septal compartment in all patients.

**Conclusion:**

According to the results, tendon of flexor carpi radialis crosses the wrist region superficial to the carpal tunnel. Thus, tendon of flexor carpi radialis doesn’t have any effect on the carpal tunnel syndrome. Further cadaveric studies would be useful for identifying the contents of the carpal tunnel and morphological organization of the wrist.

## 1. Introduction

The antebrachial fascia, anteriorly, is continuous superficially as palmar carpal ligament and distally and deeply as a strong fibrous band; the flexor retinaculum (transverse carpal ligament) [1,2]. At the medial aspect of the wrist, ulnar artery and nerve pass through a tunnel between these ligaments, which is termed as the ulnar tunnel (guyon canal) [3]. The roof and floor of the canal is bordered by palmar carpal ligament and transvers carpal ligament, respectively [1–3]. Thick flexor retinaculum also encloses the carpal groove superiorly and forms a passageway on the palmar side of the wrist [4,5]. The carpal tunnel is an osseo-fibrous tunnel which is bordered superiorly by transverse carpal ligament and the base consist of the hook of hamate, triquetrum, and pisiform bones medially; scaphoid and the trapezium bones laterally [4,6,7]. The tendons of the flexor muscles of the fingers and the median nerve pass through the tunnel [2,4,5,7]. Most of the textbooks mentioned that 10 structures traverse the tunnel including the 9 tendons which are flexor digitorum superficialis and profundus, and palmaris longus and the median nerve [1,4,5,7,8]. On the anterior surface of the wrist, the flexor retinaculum and palmar carpal ligament held the tendons of the flexor muscles in the tunnel [9]. The tendon of the flexor carpi radialis (FCRt) passes through a vertical groove on the trapezium within its own tunnel at the wrist region, which is separated from the carpal tunnel by the deep portion of the transverse carpal ligament and inserts on the palmar surface of the base of the second and third metacarpal [1,8–11]. In addition to this well-known information, it is also mentioned that FCRt found within the carpal tunnel in its own tunnel [8]. Previous studies have focused on describing the carpal tunnel’s borders and related structures by using different methods to identify the safe zones for carpal tunnel release [12–14]. MRI and ultrasound methods are used for identification of the course of FCRt and diagnosing the wrist pathologies [15–18]. Carpal tunnel syndrome is the most common entrapment neuropathy characterized by tingling, burning, pain, and paresthesia in the first 3 radial digits and radial half of the forth digit due to the compression of the median nerve in the carpal tunnel [19–22]. Although it has been widely studied, while mentioning the contents of the carpal tunnel, differences could be seen among the textbooks [1,2,8,23–26]. There is no conflict about the fact that the tendons of flexor digitorum superficialis, flexor digitorum profundus, flexor pollicis longus, and the median nerve are located inside the carpal tunnel. The presence of the tendon of flexor carpi radialis in the tunnel could be confusing. The aim of this study is to reveal the relationship between FCRt and carpal tunnel. Thus, the borders and contents of the carpal tunnel could be described easily. Results of this study could make an important contribution to the literature and support the diagnostic studies about carpal tunnel syndrome.

## 2. Materials and methods

This study was carried out in pursuit of receiving the ethical approval from the local ethics committee (GO 17/887-15) and completed according to the principles of the Helsinki Declaration.

### 2.1. Participants

Retrospectively, 118 (74 female and 44 male) wrist regions MRI scans were evaluated. None of the patients had surgery or trauma history. Patients with wrist pathology were excluded. The mean age of the patients was 35.7 (range: 9–74). In addition, 3 fresh-frozen upper limb cadavers were dissected to demonstrate the course of the flexor carpi radialis tendon.

### 2.2. Image acquisition

Wrist MRI evaluations were completed using any of three 1.5 Tesla scanners in the radiology department (SymphonyTim, Siemens Healthcare; Achieva, Philips Healthcare; Signa HDxt, GE Healtcare). MRI protocols were performed while patients were in the supine position. The radiology department’s routine MRI protocol consisted of coronal, sagittal, and transverse T2-weighted spin-echo and sagittal and transverse T1-weighted spin-echo with 3.0–3.5 mm slice thickness. All patients’ MRI series were obtained from the Picture Archiving and Communication System (PACS) at authors’ university hospital. All evaluations were completed by a 20 year experienced anatomy professor, a 15-year experienced anatomy specialist, a 6-year experienced anatomy specialist and a 20-year experienced orthopedic associated professor by using Osirix-Lite version 9 (Pixmeo, SARL, Switzerland).

### 2.3. Parameters 

To standardize the evaluation, the series of axial sections from the level of pisiform bone were examined. First location of the FCRt, proximally to the carpal tunnel was detected. Afterward the course of the tendon was followed till it passed through the carpal tunnel and its location according to the flexor retinaculum was observed. While dissecting the fresh-frozen cadavers, first incision was applied vertically from the anterior surface of the middle of the forearm to the proximal part of the palmar surface of the hand. The skin and superficial connective tissues were deviated carefully to view the muscle groups of the anterior compartment of forearm and the flexor retinaculum. The entire course of tendons of the flexor muscles were evaluated.

## 3. Results

**Figure 1:**
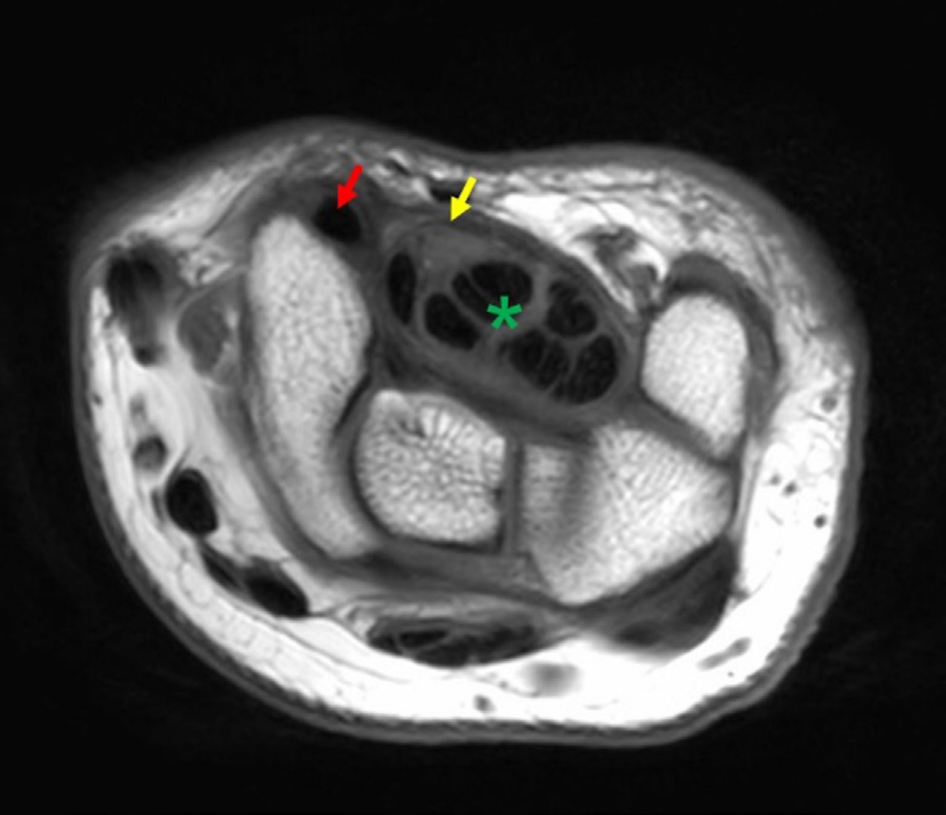
An MRI section demonstrating flexor carpi radialis tendon’s relation with the carpal tunnel. Red arrow; the tendon of flexor carpi radialis. Yellow arrow; flexor retinaculum. Green asterix; tendons of flexor dig

**Figure 2:**
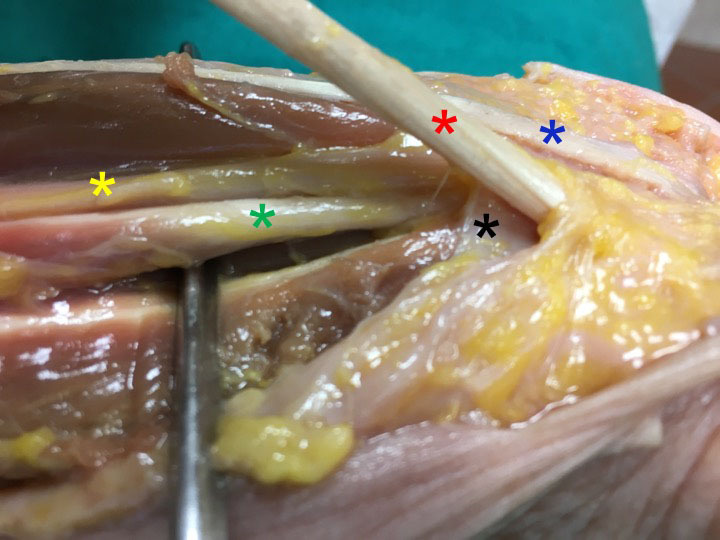
Dissection of the wrist joint. Black asterix; flexor retinaculum. Red asterix; tendon of flexor carpi radialis. Blue asterix; tendon of palmaris longus. Yellow asterix; median nerve. Green asterix; tendon of flexor pollicis longus muscle.

In all MRI series the flexor tendons of the digits and median nerve were located below the flexor retinaculum while the FCRt was located above the flexor retinaculum within its own septal compartment (Figure 1). Likewise, the cadaveric dissections also executed that the flexor tendons of digits and median nerve were covered by flexor retinaculum within the carpal tunnel and, a completely different and separated compartment of the FCRt was found above the flexor retinaculum (Figure 2 and Figure 3). 

## 4. Discussion

At wrist region distal continuation of the antebrachial fascia forms superficially the palmar carpal ligament and deeply the flexor retinaculum (transverse carpal ligament) [1,2]. Between palmar carpal ligament and flexor retinaculum guyon canal is found which includes ulnar artery and nerve [3]. Strong fibrous flexor retinaculum forms the roof of the carpal tunnel [7,12]. It is important to define the flexor retinaculum’s boundaries precisely to identify the contents of carpal tunnel. Furthermore, this will lead to creating common terminology between the anatomy and clinical sciences. The FCR muscle is found at the anterior compartment of the forearm and mainly flexes and to a lesser extend radially rotates the wrist [16]. Although its morphological and clinical importance has been widely studied, there is still no consensus about its relationship with the carpal tunnel [1,2,8,9,23–26]. Most of the textbooks mentioned that 10 structures traverse the tunnel including the 9 tendons including flexor digitorum superficialis and profundus, and palmaris longus and the median nerve [1,4,5,7,8]. However, some other textbooks mention that the FCRt found in the tunnel [8]. 

Morphological studies have demonstrated that the FCRt was not included in the contents of the carpal tunnel [6,16,27–29]. Chamas et al. described as FCRt forms the lateral border of the carpal tunnel in a morphological study that focused on the explaining the boundaries of carpal tunnel [7]. The FCRt is covered by a fibro-osseous tunnel which is adjacent to the flexor retinaculum of the carpal tunnel [4,10]. This tunnel is formed proximally by the scaphoid, the flexor retinaculum, and a vertical retinacular septum and, distally, by trapezium, again the flexor retinaculum, and the vertical retinacular septum [10]. This vertical retinacular septum separates the FCRt from the carpal tunnel [10,16,30]. Also, it is usually used as donor in tendon transfers and reconstructive surgeries of the forearm [22,31]. Due to this proximity, any disorder like tenosynovitis of the FCRt may imitate carpal tunnel syndrome [10]. Therefore, FCR tenosynovitis should be considered in the differential diagnosis of carpal tunnel syndrome and a detailed examination should be performed.

According to the results of this study, the FCRt crosses the wrist superficial to the carpal tunnel. Thus, the FCRt may not be included as a factor in the etiology of the carpal tunnel syndrome but the close relationship must be in mind that the pathologies may imitate the carpal tunnel syndrome. Further multidisciplinary studies would be useful for identifying the contents of the carpal tunnel, the morphological organization of the wrist, and the biomechanical etiologies and mechanisms of the carpal tunnel syndrome.

Although, the well-known theoretical information about the carpal tunnel, there is still no consensus about situation of the FCRt in major anatomy textbooks. Therefore, this study was demonstrated the precise situation of the tendon of the flexor carpi radialis with the carpal tunnel. Results verified that the FCRt does not pass through the carpal tunnel, but it passes through its own compartment that is formed by the flexor retinaculum. The main limitation of the present study was the lack of cadaveric measurements due to the insufficient number of specimens. Moreover, a histological investigation should be performed to understand the course of the flexor retinaculum. 

## Informed consent

This study was carried out in pursuit of receiving the ethical approval from the local ethics committee (GO 17/887-15) and completed according to the principles of the Helsinki Declaration.
